# Rate and risk factors of kidney function decline among South Asians with type 2 diabetes: analysis of the CARRS Trial

**DOI:** 10.1136/bmjdrc-2024-004218

**Published:** 2024-08-16

**Authors:** Kavita Singh, Dimple Kondal, Ram Jagannathan, Mohammed K Ali, Dorairaj Prabhakaran, K M Venkat Narayan, Shuchi Anand, Nikhil Tandon, CARRS Trial Investigators, Premlata K Varthakavi

**Affiliations:** 1 Public Health Foundation of India, New Delhi, Delhi, India; 2 Heidelberg Institute of Global Health, Heidelberg University, Heidelberg, Germany; 3 Centre for Chronic Disease Control, New Delhi, Delhi, India; 4 Emory University School of Medicine, Atlanta, Georgia; 5 Emory Global Diabetes Research Center, Woodruff Health Sciences Center and Emory University, Atlanta, Georgia, USA; 6 Family and Preventive Medicine, Hubert Department of Global Health, Emory University, Atlanta, Georgia, USA; 7 Public Health Foundation of India, Gurgaon, India; 8 Rollins School of Public Health, Atlanta, Georgia, USA; 9 Stanford University, Stanford, California, USA; 10 All India Institute of Medical Sciences, New Delhi, India

**Keywords:** Function, Diabetes Mellitus, Type 2, Kidney Function Tests, Diabetes Complications

## Abstract

**Introduction:**

People with diabetes are at risk of developing chronic kidney disease. However, limited data are available to quantify their risk of kidney function decline in South Asia. This study evaluates the rate and predictors of kidney function decline among people with type 2 diabetes in South Asia.

**Research design and methods:**

We analyzed data from the Centre for Cardiometabolic Risk Reduction in South Asia (CARRS) Trial to quantify the rate of decline in estimated glomerular filtration rate (eGFR) in people with type 2 diabetes (n=1146) over 2.5 years of follow-up. The CARRS Trial evaluated a multicomponent intervention of decision-supported electronic health records and non-physician care coordinator to improve diabetes management at 10 diabetes clinics in India and Pakistan. We used linear mixed models to estimate eGFR slope among all participants and tested the association of eGFR slope with demographic, disease-related, and self-care parameters, accounting for randomization and site.

**Results:**

The mean age of participants was 54.2 years, with a median duration of diabetes of 7.0 years (IQR: 3.0 - 12.0) and median CKD-EPI (Chronic Kidney Disease Epidemiology Collaboration) eGFR of 83.6 (IQR: 67.7 to 97.9) mL/min/1.73 m^2^. The overall mean eGFR slope was −1.33/mL/min/1.73 m^2^/year. There were no differences in the eGFR slope by treatment assignment to intervention versus usual care. In the adjusted regression model, pre-existing diabetic retinopathy (slope difference: −2.11; 95% CI: −3.45 to –0.77), previous cardiovascular disease (−1.93; 95% CI: −3.45 to –0.40), and statins use (−0.87; 95% CI: −1.65 to –0.10) were associated with faster eGFR decline.

**Conclusions:**

People with diabetes receiving care at urban diabetes clinics in South Asia experienced annual eGFR decline at two times higher rate than that reported from other contemporary international diabetes cohorts. Risk factors for faster decline were similar to those previously established, and thus care delivery models must put an additional emphasis on kidney protective therapies among subgroups with microvascular and macrovascular diabetes complications.

**Trial registration number:**

NCT01212328.

**WHAT IS ALREADY KNOWN ON THIS TOPIC**

- A large proportion of patients with diabetes in South Asia are at risk of developing chronic kidney disease. However, limited data are available which quantified this risk of kidney function decline or identified the risk factors of decline in kidney function amenable to modification.

**WHAT THIS STUDY ADDS**

- The current study evaluates the rate and predictors of kidney function decline among people with type 2 diabetes in South Asia.
- Patients with poorly controlled type 2 diabetes receiving care at tertiary care urban diabetes clinics in South Asia experienced annual kidney function decline (as measured by estimated glomerular filtration rate) at a rate two times higher than other populations.

**HOW THIS STUDY MIGHT AFFECT RESEARCH, PRACTICE OR POLICY**

- Data indicate that prior CVD and diabetic retinopathy were associated with faster decline in people with type 2 diabetes in South Asia.
- Thus, diabetes care delivery models must emphasize on kidney protective therapies among subgroups with microvascular and macrovascular complications.

## Introduction

Diabetes is a leading cause of chronic kidney disease (CKD), and the coexistence of these conditions multiplies the risk of premature cardiovascular events and mortality.^1^ Around 20%–40% of individuals with diabetes develop diabetic nephropathy, which is a progressive kidney disease with high risks for reaching end-stage kidney disease.^2^ Prior studies have reported more rapid kidney function decline among patients with poorer glycemic control and higher blood pressure (BP).^3^

In addition to the differences in individual level risk factor management contributing to variation in the prevalence of diabetic nephropathy, its incidence may also vary by race, ethnicity, or geography.^4^ These factors may further alter susceptibility to kidney disease through genetic, environmental, or health system-based factors.^5^ In the Pima Indian population, for example, 60% of patients with diabetes and proteinuria developed end-stage kidney disease over 15 years, compared with 17% of patients in a European white population from the Mayo Clinic.^6^ Differences in healthcare practices such as annual preventative screening for albuminuria or prescription of renin–angiotensin inhibitors, whether driven by resource constraints or gaps in implementation, may further affect risk for incident kidney disease among patients with diabetes.^4 7 8^

South Asia faces a disproportionately high burden of diabetes with one of the highest prevalence rates of diabetes globally, and South Asians tend to manifest diabetes at a much younger age compared with Western populations. As a result, a significant proportion of patients with diabetes in the region are at risk of developing CKD.^9^,^11^ Limited data are available which have quantified this risk or identified the risk factors amenable to modification. We leveraged existing data from the Centre for Cardiometabolic Risk Reduction in South Asia (CARRS) Trial to evaluate the rate and risk factors of kidney function decline among South Asian patients with poorly controlled type 2 diabetes. The CARRS Trial was a randomized trial that evaluated the effectiveness of a clinic-based multicomponent quality improvement strategy versus usual care in 1146 patients with type 2 diabetes in India and Pakistan.^12 13^ We used data spanning 2.5 years of follow-up from the CARRS Trial to describe changes in estimated glomerular filtration rate (eGFR) and identify predictors of kidney function decline among patients with type 2 diabetes in India and Pakistan.

## Methods

### Study design and setting

The rationale and design of the CARRS Trial were reported previously.^14^ Briefly, the CARRS Trial evaluated the effectiveness of a multicomponent quality improvement strategy versus usual care in 10 academic tertiary care centers located in India and Pakistan. Patients were included if they had evidence of hemoglobin A1c (HbA1C) measurements≥8% and systolic BP≥140 mm Hg or low-density lipoprotein (LDL) cholesterol level≥130 mg/dL. The CARRS Trial multicomponent quality improvement intervention comprised of a non-physician care coordinator working to improve patient self-care and decision-supported electronic health records to enhance physician’s responsiveness to timely treatment modification.^13^ Data from both treatment groups (n=1146) were combined and included in this analysis to investigate the correlates of eGFR decline in patients with diabetes.

### Outcomes

We examined eGFR slopes and factors associated with eGFR slope in the entire CARRS Trial cohort. This analysis included and reported data from all study participants and their baseline, annual, and end of study visit measurements. The end of study visits occurred between 24 and 36 months after randomization. In sensitivity analyses, we also evaluated eGFR decline> −2.0 mL/min/1.73m^2^/year (vs not) as an outcome indicating rapid kidney function decline.

### Exposures and covariates

Data collection at baseline and annual visits included assessing BP, HbA1c, LDL, cholesterol, serum creatinine, and urine albumin-to-creatinine ratio measurements. All blood samples were analyzed by local participating site laboratories with external quality assurance certification. BP was measured with an electronic device (Omron T9P). Open-ended questions about macrovascular and microvascular complications, adverse events, serious adverse events, or hospitalizations were self-reported by participants. Macrovascular complications were defined as any one of the following: coronary heart disease (myocardial infarction or unstable angina); chronic stable angina; revascularization (angioplasty (percutaneous coronary intervention)/coronary artery by-pass surgery), stroke, or peripheral vascular disease. Microvascular complications were defined as any of the following: diabetic retinopathy, neuropathy, or nephropathy. Participant’s age was grouped into 35–49, 50–64, and ≥65 years. Self-reported education status was categorized as up to primary school, secondary school, and graduate and above. The duration of diabetes was categorized as ≤7 years and >7 years, based on the median duration of the cohort. The CKD-EPI 2009 equation was used to calculate the eGFR values.^15^ We preferentially used the CKD-EPI 2009 equation rather than CKD-EPI 2021 equation because in our previous analyses evaluating association with mortality, eGFR estimates from CKD-EPI 2009 aligned with the KDIGO eGFR categories and expected mortality risk among South Asians^16^ .

### Statistical analysis

The baseline characteristics of all enrolled participants were presented as means (SD) or proportions (%). The baseline characteristics were reported overall and by sex. To estimate the individual eGFR slopes over time, we used linear mixed effect models with random intercepts and random slopes.^17^ This model included eGFR as a response variable, a fixed covariate of the time measurements, a fixed categorical effect of treatment, and taken after randomization with random intercept and time terms. The model enabled individual participant slopes to vary by random effects of intercept and time. An unstructured covariance matrix was used to model the correlation of random effects. The percentile of eGFR slope was calculated and was reported with 95% CI. The linear mixed effect model was used to estimate the difference in eGFR slopes by baseline demographic, self-reported disease complications, anthropometry, medication use, BP, and laboratory measurements. In the linear mixed effect model, covariates included were time, treatment, interaction of respective covariate with time, and site (fixed effect). The parameter for the treatment-by‐covariate interaction represents the difference in average eGFR slope among the covariate group. A common unstructured covariance structure was used to model the withinpatient errors. The adjusted models for BMI and waist circumference were done separately.

We also performed the log-binomial regression analysis to estimate the factors associated with eGFR slope greater than −2.0 mL/min/1.73 m^2^/year vs less than −2.0 mL/min/1.73 m^2^/year. The factors included were baseline demographic, self-reported disease complications, anthropometry, medication use, BP, and laboratory measurements.

All the analyses were performed using STATA V.17.0 (College Station, Texas, USA).

## Results

### Demographic characteristics of the study participants

A total of 1146 participants were included in the analysis (575 in intervention and 571 in control arm) with mean (SD) age of 54.2 (9.2) years. 54% of participants were women; the cohort median duration of diabetes was 7.0 (25th–75th percentile, 3.0–12.0) years and median eGFR was 83.6 (25th–75th percentile, 67.7–97.9) mL/min/1.73 m^2^. Demographic and clinical characteristics of study participants at baseline stratified by sex are presented in table 1.

**Table 1:** Baseline characteristics of the participants by sex

	Overall	Female	Male	P value
N	1146	619	527	
Age, years, mean (SD)	54 (9.2)	54 (9)	54 (9.4)	0.96
Age category				
35–44	185 (16.1%)	95 (15.3%)	90 (17.1%)	0.67
45–64	802 (70.0%)	435 (70.3%)	367 (69.6%)	
≥65	159 (13.9%)	89 (14.4%)	70 (13.3%)	
Education				
Up to primary	337 (29.4%)	259 (41.8%)	78 (14.8%)	<0.001
Secondary	499 (43.5%)	237 (38.3%)	262 (49.7%)	
College graduate and above	303 (26.4%)	119 (19.2%)	184 (34.9%)	
Missing	7 (0.6%)	4 (0.6%)	3 (0.6%)	
Duration of diabetes (years), median (IQR)	7 (3–12)	8 (4–12)	6 (3–13)	0.19
Duration of diabetes				
<7 years	537 (46.9%)	275 (44.4%)	262 (49.7%)	0.076
≥ years	595 (51.9%)	336 (54.3%)	259 (49.1%)	
Missing	14 (1.2%)	8 (1.3%)	6 (1.1%)	
CVD	78 (6.8%)	35 (5.7%)	43 (8.2%)	0.093
PVD	66 (5.8%)	40 (6.5%)	26 (4.9%)	0.27
Retinopathy	100 (8.7%)	48 (7.8%)	52 (9.9%)	0.21
Neuropathy	380 (33.2%)	210 (33.9%)	170 (32.3%)	0.55
Current smoker	34 (3.0%)	2 (0.3%)	32 (6.1%)	<0.001
BMI (kg/m^2^), mean (SD)	27 (5)	28 (4.9)	26 (5)	<0.001
BMI (kg/m^2^)				
<18.5	13 (1.1%)	5 (0.8%)	8 (1.5%)	<0.001
18.5–22.9	171 (14.9%)	68 (11.0%)	103 (19.5%)	
23.0–24.9	183 (16.0%)	72 (11.6%)	111 (21.1%)	
≥25	779 (68.0%)	474 (76.6%)	305 (57.9%)	
Hemoglobin A1c, % mean (SD)	9.9 (1.6)	10 (1.6)	9.9 (1.6)	0.46
Hemoglobin A1c %category				
<9	383 (33.4%)	198 (32.0%)	185 (35.1%)	0.26
≥9	763 (66.6%)	421 (68.0%)	342 (64.9%)	
Systolic blood pressure (mm Hg), mean (SD)	143.3 (19.4)	141.8 (19.9)	145.0 (18.8)	0.007
Systolic blood pressure (mm Hg) category				
<140	350 (30.5%)	200 (32.3%)	150 (28.5%)	0.16
≥140	796 (69.5%)	419 (67.7%)	377 (71.5%)	
LDL-c (mg/dL), mean (SD)	122.4 (36.9)	125.1 (38.3)	119.2 (34.9)	0.007
LDL-c (mg/dL) category				
<130	604 (52.7%)	312 (50.4%)	292 (55.4%)	0.091
≥130	542 (47.3%)	307 (49.6%)	235 (44.6%)	
Waist circumference, mean (SD)	96 (11)	97 (12)	95 (11)	0.008
Waist circumference				
<80 cm (women), <90 cm (men)	185 (16.1%)	29 (4.7%)	156 (29.6%)	<0.001
≥80 cm (women), ≥ 90 cm (men)	961 (83.9%)	590 (95.3%)	371 (70.4%)	
eGFR (CKD-EPI 2009) equation, mean (SD)	83 (21)	80 (21)	86 (20)	<0.001
eGFR (CKD-EPI 2009) equation, median (IQR)	84 (68, 98)	81 (65, 97)	87 (74, 100)	<0.001
Creatinine, mg/dL, mean (SD)	0.98 (0.68)	0.93 (0.83)	1 (0.43)	0.005
Creatinine, mg/dL, median (IQR)	0.9 (0.8–1.1)	0.8 (0.7–1)	1 (0.9–1.1)	<0.001
Urinary albumin–creatinine ratio (IQR), mg/g, median(IQR)	66.6 (283.6)	68.8 (350.7)	64.1 (174.9)	0.78
<30	765 (66.8%)	428 (69.1%)	337 (63.9%)	0.091
30–299.99	338 (29.5%)	173 (27.9%)	165 (31.3%)	
≥300	43 (3.8%)	18 (2.9%)	25 (4.7%)	
EuroQol-5D Score, mean (SD)	70 (20)	68 (20)	72 (19)	<0.001
EuroQol-5D Score, median (IQR)	70 (50–89)	70 (50–85)	75 (60–90)	<0.001
Medication use, %				
Oral hypoglycemic agents	1083 (94.5%)	592 (95.6%)	491 (93.2%)	0.068
Metformin	610 (53.2%)	326 (52.7%)	284 (53.9%)	0.68
Sulfonylurea	419 (36.6%)	235 (38.0%)	184 (34.9%)	0.29
Blood pressure-lowering drugs	714 (62.3%)	479 (77.4%)	356 (67.6%)	<0.001
ACEi/ARB	317 (27.7%)	135 (25.6%)	182 (29.4%)	0.153
Beta-blockers	110 (9.6%)	68 (11.0%)	42 (8.0%)	0.084
Calcium channel blockers	116 (10.1%)	67 (10.8%)	49 (9.3%)	0.39
Thiazide	58 (5.1%)	31 (5.0%)	27 (5.1%)	0.93
Statins	679 (59.2%)	378 (61.1%)	301 (57.1%)	0.17

Data are presented as mean (SD) or median (IQR Q1–Q3).

ACEi/ARB, ACE inhibitors/angiotensin II receptor blockers ; BMI, body mass index; eGFR, estimated glomerular filtration rate; LDL-c, low-density lipoprotein cholesterol.

Compared with women, men were more likely to have received graduate degrees, used tobacco, and had higher systolic BPs, but had lower BMI and LDL cholesterol. Baseline eGFR was higher among men than women. eGFR slope decline over the study period.

**Figure 1:**
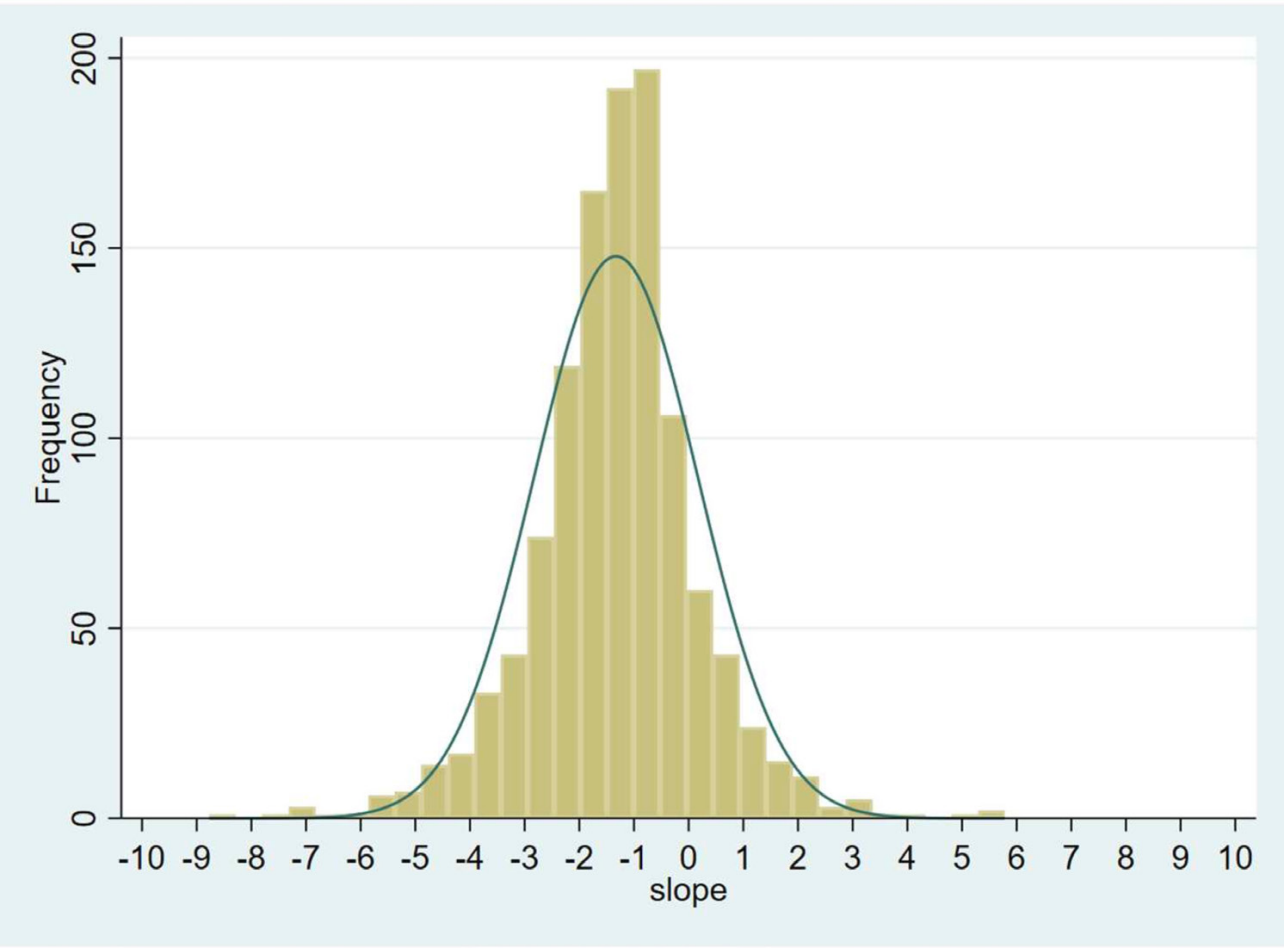
Distribution of estimated glomerular filtration rate slope in the entire Centre for Cardiometabolic Risk Reduction in South Asia Trial cohort over the study period.

Figure 1 shows the distribution of eGFR slope. The median eGFR slope was −1.26 (95% CI: −1.34 to –1.19) mL/min/1.73 m^2^/year. Mean eGFR slope was −1.33 (95% CI: −1.699 to –0.96) mL/min/1.73 m^2^/year. Table 2 shows the distribution of eGFR slopes in percentiles. 27% and 10% experienced an eGFR slope of > −2 and −3 /mL/min/1.73 m^2^/year, respectively.

**Table 2:** Percentile for eGFR slope

Percentile	Centile (95% CI)
10	−3.08 (−3.28 to −2.89)
20	−2.33 (−2.45 to −2.18)
30	−1.90 (−2.00 to −1.83)
40	−1.59 (−1.69 to −1.47)
50	−1.26 (−1.34 to −1.19)
60	−0.99 (−1.08 to −0.93)
70	−0.74 (−0.82 to −0.66)
80	−0.37 (−0.48 to −0.26)
90	0.37 (0.17 to 0.63)

eGFR, estimated glomerular filtration rate.

**Figure 2:**
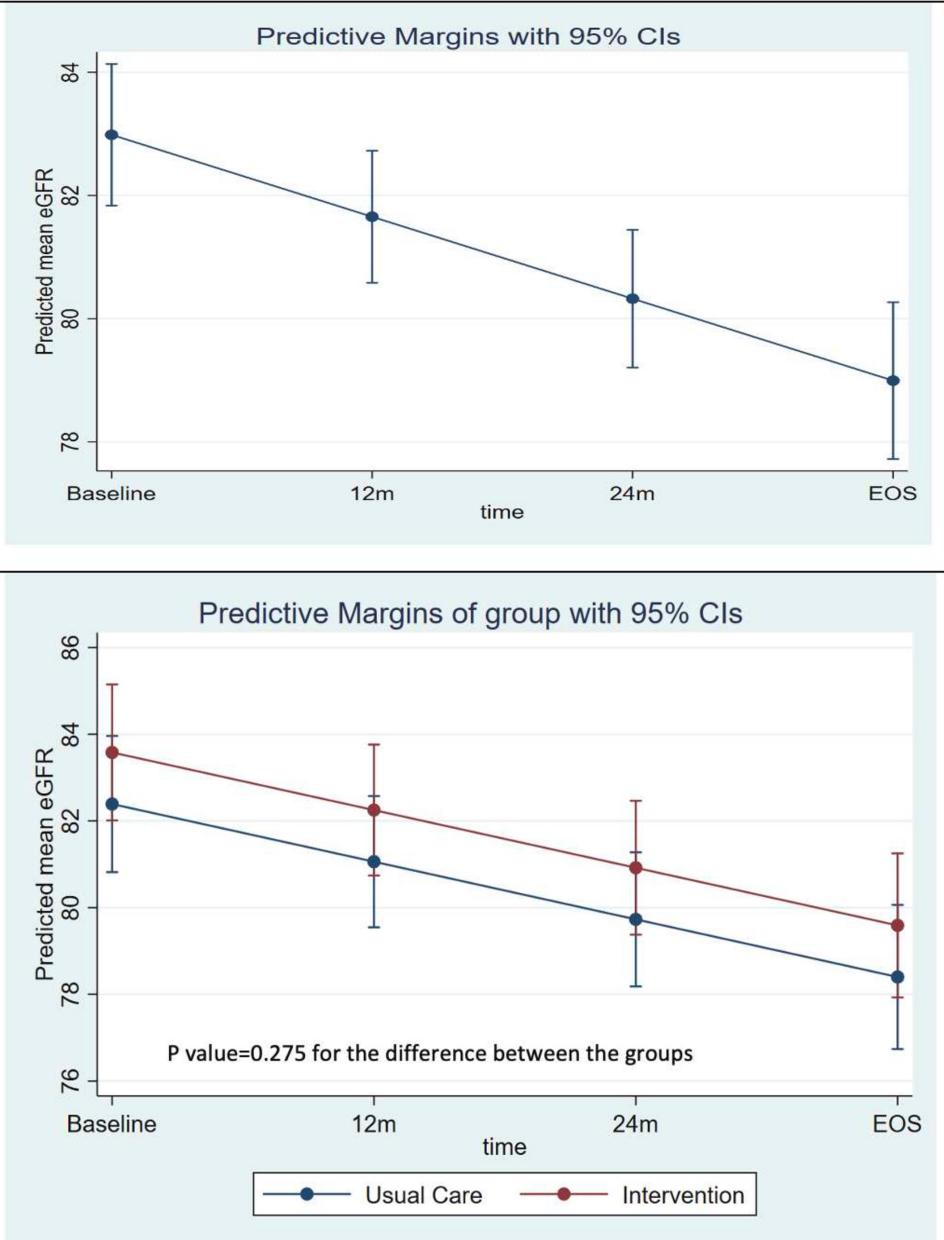
(A) Predicted mean eGFR over the study period. (B) Predicted mean eGFR over the time period by treatment group. eGFR, estimated glomerular filtration rate.

Overall, the mean predicted eGFR at baseline was 83.0 (95% CI: 81.8 to 84.1) mL/min/1.73 m^2^/year, and at trial end after 2.5 years of follow-up, the predicted mean eGFR was 79.0 (95% CI: 77.7 to 80.3) mL/min/1.73 m^2^/year (figure 2). There were no differences in eGFR slope by treatment arm (predicted mean eGFR over the time period by treatment group is shown in (figure 2). The mean eGFR values overall and by treatment groups are provided in online supplemental etable 1.

### Risk factors for eGFR decline

Table 3 presents the baseline risk factors predicting decline in eGFR slope. Pre-existing cardiovascular disease, presence of diabetic retinopathy, and statins use were associated with significantly faster decline in eGFR, compared with patients lacking these features at baseline. There were no significant differences in eGFR slopes by age, sex, education, smoking, BMI, waist circumference, and baseline HbA1c, LDL cholesterol and systolic BP. Online supplemental etable 2 shows the factors associated with decline in eGFR slope greater than −2.0 mL/min/1.73 m^2^ (n=313) vs less than −2.0 units (n=833) decline in eGFR. These results are consistent with the primary analysis reported in table 3 (eg, diabetes duration is associated with higher risk for eGFR> −2.0 mL/min/1.73 m^2^ vs not), with the added findings that pre-existing CVD, prescription of statins, and insulin at baseline were significantly associated with a rapid decline in eGFR in our unadjusted model (p<0.05). These associations were attenuated in the regression model adjusted for treatment group and each of the covariates listed in the model. However, there was a consistent association with UACR≥300 mg/g (vs not) resulting in higher risk for eGFR> −2.0 mL/min/1.73 m^2^ vs not (risk ratio 1.7 (95% CI 1.07 to 2.82) in adjusted models).

**Table 3:** Association of eGFR slope with the baseline risk factors

	Unadjusted difference (95% CI)		Adjusted^*^ difference (95% CI)		Adjusted† difference (95% CI)	
Age (ref=35–44 years)						
45–64 years	−0.442	(−1.469 to 0.584)	−0.396	(−1.457 to 0.664)	−0.393	(−1.454 to 0.667)
≥ 65 years	−0.36	(−1.749 to 1.030)	−0.485	(−1.919 to 0.948)	−0.480	(−1.913 to 0.954)
Sex						
Female (ref=men)	0.0084	(−0.733 to 0.750)	−0.143	(−0.904 to 0.619)	−0.145	(−0.906 to 0.616)
Education						
Up to primary school	−0.397	(−1.396 to 0.600)	−0.282	(−1.302 to 0.738)	−0.284	(−1.303 to 0.736)
Secondary school	−0.285	(−1.182 to 0.611)	−0.097	(−1.012 to 0.819)	−0.099	(−1.015 to 0.816)
College graduate or above (ref)						
Duration of diabetes (ref≤7 years)						
≥7 years	−0.727	(−1.472 to 0.0188)	−0.737	(−1.496 to 0.021)	−0.736	(−1.495 to 0.023)
Comorbidities (ref=no)						
Previous cardiovascular disease	−2.148**	(−3.624 to –0.672)	−1.931	(−3.452 to –0.409)	−1.927	(−3.448 to –0.405)
Previous peripheral vascular disease	−1.457	(−3.068 to 0.155)	−1.504	(−3.177 to 0.17)	−1.497	(−3.171 to 0.176)
Previous retinopathy	−2.087**	(–3.391 to –0.783)	−3.302	(−7.091 to 0.487)	−2.109	(−3.448 to –0.771)
Previous neuropathy	−0.525	(−1.310 to 0.260)	−2.303	(−4.715 to 0.109)	−0.481	(−1.287 to 0.325)
Current smoker (ref=no)						
Yes	−1.224	(−3.297 to 0.849)	−1.253	(−3.355 to 0.848)	−1.249	(−3.35 to 0.852)
Body mass index (kg/m^2^)						
<18.5						
18.5–22.9	−0.941	(−4.478 to 2.596)	−0.591	(−4.179 to 2.996)	na	
23–24.9	−0.631	(−4.156 to 2.894)	−0.498	(−4.072 to 3.075)		
≥25	−0.337	(−3.771 to 3.097)	−0.184	(−3.663 to 3.296)		
HbA1c at baseline (ref<9.0%)						
≥9%	−0.722	(−1.499 to 0.055)	−0.716	(−1.514 to 0.082)	−0.716	(−1.515 to 0.082)
LDL at baseline (ref<130 mg/dL)						
≥130 mg/dL	−0.615	(−1.354 to 0.124)	−0.639	(−1.398 to 0.119)	−0.639	(−1.398 to 0.119)
SBP at baseline (ref<140 mm Hg)						
≥140 mm Hg	0.324	(−0.471 to 1.120)	0.266	(−0.55 to 1.083)	0.266	(−0.55 to 1.082)
Waist circumference (ref, <80 cm (women), <90 cm (men)						
≥80 cm (women), ≥90 cm (men)	−0.301	(−1.300 to 0.697)	na		−0.431	(−1.455 to 0.593)
Blood pressure lowering medication (ref=no)						
Yes	0.603	(−0.160 to 1.366)	0.684	(−0.102 to 1.469)	0.684	(−0.102 to 1.469)
Statins (ref=no)						
Yes	−0.815*	(−1.566 to –0.0636)	−0.877	(−1.65 to –0.105)	−0.875	(−1.648 to –0.103)
Hypoglycemic agents (ref=no)						
Yes	−1.115	(−2.802 to 0.573)	−1.332	(−3.055 to 0.39)	−1.328	(−3.051 to 0.394)
ACEi/ARB						
Yes	0.334	(−0.488 to 1.157)	0.251^‡^	(−0.592 to 1.094)	0.251^§^	(−0.591 to 1.094)
Albumin creatinine ratio (ref<30 mg/g)						
30–299.9 mg/g	−0.457	(−1.291 to 0.375)	−0.434	(−1.273 to 0.405)	−0.439	(−1.278 to 0.401)
≥300 mg/g	−1.442	(−3.298 to 0.413)	−1.390	(−3.238 to 0.458)	−1.376	(−3.225 to 0.472)

* Adjusted for the treatment group and each of the other covariates listed in the model (excluding waist circumference).

† Adjusted for the treatment group and each of the other covariates listed in the model (excluding body mass index).

‡ Adjusted for the treatment group and each of the other covariates listed in the model (excluding waist circumference and BP medication).

§ Adjusted for the treatment group and each of the other covariates listed in the model (excluding body mass index and BP medication).

ACEi/ARB, ACE inhibitors/angiotensin II receptor blockers; eGFR, Estimated glomerular filtration rate; HbA1c, hemoglobin A1c; LDL, low-density lipoprotein; ref, reference; SBP, systolic blood pressure.

## Discussion

Using data from the CARRS Trial cohort, we demonstrate that South Asians with diabetes accessing care at tertiary care centers are experiencing eGFR loss at ~1.3 mL/min/1.73 m^2^ annually. Loss of kidney function was more rapid among patients with diabetes with longer disease duration, pre-existing cardiovascular disease and pre-existing diabetic retinopathy. A majority (>80%) of the cohort was experiencing some loss of kidney function, and approximately a quarter of patients were experiencing rapid eGFR loss (at >2 mL/min/1.73 m^2^ annually). Given the context that participants in our study were accessing care at premier regional institutions, these findings indicate that kidney health protection requires urgent emphasis in diabetes clinics in South Asia.

The mean eGFR slope observed in this study was almost twofold higher than the mean eGFR decline reported in other populations. For example, the ADVANCE ON Trial^18^ analysis of 8879 participants from Caucasian, Black, and Hispanic backgrounds, with similar duration of diabetes (mean 7.8 years) and similar or lower mean eGFR (75 mL/min/1.73 m^2^), the mean eGFR slope over 20 months of follow-up was −0.63 mL/min/1.73 m^2^/year. Furthermore, eGFR decline faster than the lowest quartile (< −1.6 mL/min/1.73 m^2^) was associated with 30% higher risk for a combined outcome of major cardiovascular, macrovascular events or all-cause mortality.^19^ However, in the EMPA-REG OUTCOME Trial, the standard of care group experienced eGFR slope of −1.4 mL/min/1.73 m^2^/year, similar to the one we observed in our study, although the duration of diabetes was longer (57% had diabetes for >10 years) among participants in this study than ours.^20^ Furthermore, a quarter of participants in the standard of care arm had pre-existing kidney disease, as defined by eGFR<60 mL/min/1.73 m^2^. A significant proportion of our cohort experienced eGFR decline at more than 3 mL/min/1.73 m^2^, a rate at which the risk for heart failure, coronary artery disease and peripheral arterial disease all increase by 30%–60%.^21^

Although we had limited exposure information on some of the hypothesized “non-traditional” risk factors for kidney dysfunction such as occupation or heat exposure, we did not observe an association between sociodemographics factors and kidney function decline in this cohort. Our study results corroborate previous research and showed that the risk of rapid eGFR decline was higher among patients with longer diabetes duration, which is also an important predictor of microvascular complications.^22 23^ For example, the Action to Control Cardiovascular Risk in Diabetes study involving 10 251 people with type 2 diabetes and baseline HbA1c or >7.5% found that patients with youngest age-of-onset or longer duration of diabetes had more rapid declines in eGFR compared with those diagnosed at middle age or those with shorter duration of diabetes.^24^ As disease duration is a non-modifiable factor, focus should be on prevention, early diagnosis, and timely treatment intensification to arrest high decline in eGFR.

The presence of any microvascular complications, primarily diabetic retinopathy, remains important predictors of rapid eGFR decline.^3 25^ The development of CKD likely worsens the existing microvascular disease (diabetic retinopathy) due to the sodium retention and disorders of calcium hemostasis that are often a result of CKD. Similarly, coexistence of prior cardiovascular disease was a strong predictor of poor eGFR decline in this study. In this study, we found relatively low utilization of ACEi/ARB in high-risk patients with poorly controlled type 2 diabetes, as less than a third of our cohort was on these class of drugs at baseline. This underscores the need to enhance the prescription of guideline-directed medical therapy and to carefully evaluate kidney function and kidney outcomes.

Previous diabetes trials have shown that intensified, multifactorial treatment slowed the progression of nephropathy and renal function loss, reducing the risk of end-stage renal disease. In this study, we did not find a statistically significant difference in mean eGFR decline between the treatment groups, which suggest that the CARRS Trial multicomponent quality improvement intervention did not influence the eGFR slope significantly between the treatment groups in the short term (2.5 years of follow-up). However, the STENO-2 Trial from Denmark involving 160 type 2 diabetes patients, in whom strict risk factor control, including strong emphasis on albuminuria-targeted therapies was evaluated, the intervention arm experienced slower eGFR decline over.^26^ Further, the progression to end-stage renal disease was lower in the intervention arm (HR 0.36, 95% CI: 0.12 to 1.05).^26^ With institution of empagliflozin therapy in addition to standard of care renin–angiotensin inhibition, the intervention arm in the EMPA-REG study experienced no decline in eGFR over (slope: 0.23 mL/min/1.73 m^2^/year).^27^ Thus, with aggressive institution of existing therapies, kidney function can be preserved in patients with diabetes. A programmatic approach to doing so in South Asia clearly necessary based on our data.

We acknowledge several important limitations in our work. First, the GFR was estimated using serum creatinine rather than measured directly, which introduces imprecision. Repeated measurements of glomerular filtration rate using inulin clearance, for example, are generally not feasible because of the high cost, participant burden, and the need for specialized technical expertise. Second, although we measured a range of potential risk factors for kidney dysfunction, we have limited data on putative “non-traditional” risk factors (eg, metabolic load, occupational history, hydration behaviors, or other potential confounding factors, such as dietary intake patterns were not assessed). The clinical setting makes it extremely difficult to obtain accurate dietary intake data and extensive data collection questionnaire affects patient retention in trials.^13^ Third, participants with poorly controlled type 2 diabetes were recruited from urban tertiary care clinics, which may not be representative of other geographic regions in South Asia. Furthermore, poorly controlled glycemia at baseline is likely to contribute to the higher rates of end-stage renal disease observed in this study. Our duration of follow-up was limited to 2.5 years, and longer duration follow-up data may elucidate additional risk factors for kidney function decline. At last, clinic-level and provider-type characteristics and health system-level factors (human resources and infrastructure) affecting kidney function markers among patients with diabetes were not analyzed, as these data were not fully captured during the trial.

This study has several strengths, including the prospective study design, and relatively large sample size. To our knowledge, this is the first study to ascertain eGFR slopes among South Asian patients with diabetes. We also identified baseline characteristics of CARRS Trial participants that were predictive of slower or rapid decline in eGFR slopes. We used standardized methods to measure biomarkers such as serum creatinine, microalbuminuria, fasting blood glucose, glycated hemoglobin, lipids and BP. The sample size was relatively large from a diverse mix of hospital settings (public, private and semiprivate), which may improve generalizability of the study findings to a larger population of poorly controlled type 2 diabetes patients. Future studies are warranted to study the impact of other important health system and environmental risk factors such as air pollution, extreme weather conditions with renal function decline in this population.

In conclusion, our study highlights important factors influencing rate and risk factors of decline in kidney function including the non-modifiable factors such as duration of diabetes or presence of complications or other comorbid conditions, which re-emphasized the importance of early diagnosis and intensive management early in the course of diabetes. Therefore, care delivery models and strategies to mitigate progression of kidney function decline must put an additional emphasis and effort on patients with longer disease duration and those having microvascular and macrovascular complications.

## Supplementary material

10.1136/bmjdrc-2024-004218 online supplemental file 1

## Data Availability

Data are available on reasonable request.

## References

[1] Thomas MC, Cooper ME, Zimmet P (2016). Changing epidemiology of type 2 diabetes mellitus and associated chronic kidney disease. Nat Rev Nephrol.

[2] Koye DN, Magliano DJ, Nelson RG (2018). The global epidemiology of diabetes and kidney disease. Adv Chronic Kidney Dis.

[3] Wang Y, Wan EYF, Mak IL (2022). The association between trajectories of risk factors and risk of cardiovascular disease or mortality among patients with diabetes or hypertension: a systematic review. PLoS One.

[4] Bhalla V, Zhao B, Azar KMJ (2013). Racial/ethnic differences in the prevalence of proteinuric and nonproteinuric diabetic kidney disease. Diabetes Care.

[5] Jagannathan R, Anand S, Hogan J (2022). Estimated glomerular filtration rate trajectories in South Asians: findings from the cardiometabolic risk reduction in South Asia study. Lancet Reg Health Southeast Asia.

[6] Nelson RG, Knowler WC, Kretzler M (2021). Pima Indian contributions to our understanding of diabetic kidney disease. Diabetes.

[7] Duru OK, Middleton T, Tewari MK (2018). The landscape of diabetic kidney disease in the United States. Curr Diab Rep.

[8] Brosius FC, Cherney D, Gee PO (2021). Transforming the care of patients with diabetic kidney disease. Clin J Am Soc Nephrol.

[9] Viswanathan V, Jamthikar AD, Gupta D (2020). Integration of estimated glomerular filtration rate biomarker in image-based cardiovascular disease/stroke risk calculator: a South Asian-Indian diabetes cohort with moderate chronic kidney disease. Int Angiol.

[10] Shah A, Kanaya AM (2014). Diabetes and associated complications in the South Asian population. Curr Cardiol Rep.

[11] Shrestha N, Gautam S, Mishra SR (2021). Burden of chronic kidney disease in the general population and high-risk groups in South Asia: a systematic review and meta-analysis. PLoS One.

[12] Ali MK, Singh K, Kondal D (2016). Effectiveness of a multicomponent quality improvement strategy to improve achievement of diabetes care goals: a randomized, controlled trial. Ann Intern Med.

[13] Masood MQ, Singh K, Kondal D (2021). Factors affecting achievement of glycemic targets among type 2 diabetes patients in South Asia: analysis of the CARRS trial. Diabetes Res Clin Pract.

[14] Shah S, Singh K, Ali MK (2012). Improving diabetes care: multi-component cardiovascular disease risk reduction strategies for people with diabetes in South Asia--the CARRS multi-center translation trial. Diabetes Res Clin Pract.

[15] Levey AS, Stevens LA, Schmid CH (2009). A new equation to estimate glomerular filtration rate. Ann Intern Med.

[16] Jagannathan R, Anand S, Kondal D (2024). Prospective Study on Kidney Dysfunction Markers and Risk for Mortality among South Asians. Kidney Int Rep.

[17] Shou H, Hsu JY, Xie D (2017). Analytic considerations for repeated measures of eGFR in cohort studies of CKD. Clin J Am Soc Nephrol.

[18] Oshima M, Jun M, Ohkuma T (2019). The relationship between eGFR slope and subsequent risk of vascular outcomes and all-cause mortality in type 2 diabetes: the ADVANCE-ON study. Diabetologia.

[19] Ohkuma T, Zoungas S, Jun M (2020). Intensive glucose-lowering and the risk of vascular events and premature death in patients with decreased kidney function: the ADVANCE trial. Diabetes Obes Metab.

[20] Zinman B, Wanner C, Lachin JM (2015). Empagliflozin, cardiovascular outcomes, and mortality in type 2 diabetes. N Engl J Med.

[21] Shlipak MG, Katz R, Kestenbaum B (2009). Rapid decline of kidney function increases cardiovascular risk in the elderly. J Am Soc Nephrol.

[22] Hoefield RA, Kalra PA, Baker PG (2011). The use of eGFR and ACR to predict decline in renal function in people with diabetes. Nephrol Dial Transplant.

[23] Ninomiya T, Perkovic V, de Galan BE (2009). Albuminuria and kidney function independently predict cardiovascular and renal outcomes in diabetes. J Am Soc Nephrol.

[24] Buyadaa O, Salim A, Morton JI (2021). Rate of decline in kidney function and known age-of-onset or duration of type 2 diabetes. Sci Rep.

[25] Perkins BA, Bebu I, Gao X (2022). Early trajectory of estimated glomerular filtration rate and long-term advanced kidney and cardiovascular complications in type 1 diabetes. Diabetes Care.

[26] Oellgaard J, Gæde P, Rossing P (2017). Intensified multifactorial intervention in type 2 diabetics with microalbuminuria leads to long-term renal benefits. Kidney Int.

[27] Wanner C, Heerspink HJL, Zinman B (2018). Empagliflozin and kidney function decline in patients with type 2 diabetes: a slope analysis from the EMPA-REG outcome trial. *J Am Soc Nephrol*.

