# Supplementary material for: Rate and risk factors of kidney function decline among South Asians with type 2 diabetes: analysis of the CARRS Trial
Source: BMJ Open Diabetes Res Care. 2024 Aug 16;12(4):e004218. doi: 10.1136/bmjdrc-2024-004218 (PMC11409274; doi:10.1136/bmjdrc-2024-004218)
Supplement: online supplemental file 1 [file bmjdrc-12-4-s001.pdf]

Supplementary Tables

eTable 1: Mean eGFR, creatinine and Urine to Albumin Creatinine ratio over the period by intervention and usual care group.

	**Overall**			**Usual Care**			**Intervention**		
**eGFR**	N	Mean (SD)	Median (IQR)	N	Mean (SD)	Median (IQR)	n	Mean (SD)	Median (IQR)
Baseline	1146	82.6 (21)	83.6 (67.7, 97.9)	571	81.3 (20.9)	82 (66.8, 97.2)	575	83.9 (21.1)	85.5 (68.7, 99.2)
12m	996	82.4 (20.7)	84.3 (68.2, 98.5)	502	81.6 (21.4)	82.9 (67.2, 97.3)	494	83.3 (19.9)	84.9 (68.7, 99.4)
24m	490	82.3 (20.8)	83.9 (69.5, 97.8)	246	83.7 (21)	87.3 (70.1, 99.2)	244	80.9 (20.5)	82.7 (68.5, 96.8)
36m	953	78.8 (21)	79.6 (64.9, 95.4)	474	79.4 (20.5)	80.8 (65.4, 95.8)	479	78.2 (21.5)	78.6 (64.5, 95.1)
**Creatinine** (mg/dL)									
Baseline	1146	0.98 (.68)	0.9 (.8, 1.1)	571	1 (.86)	0.9 (.8, 1.1)	575	.94 (.43)	0.9 (.8, 1)
12m	996	0.97 (.49)	0.9 (.8, 1)	502	1 (.64)	0.9 (.8, 1.1)	494	.93 (.24)	0.9 (.8, 1)
24m	490	0.98 (.59)	0.9 (.8, 1.1)	246	1 (.78)	0.9 (.8, 1.1)	244	.96 (.27)	0.9 (.8, 1.1)
36m	953	1 (.44)	0.91 (.8, 1.1)	474	1 (.44)	0.9 (.8, 1.1)	479	1 (.45)	0.93 (.8, 1.1)
**Urine Albumin Creatinine ratio (mg/g)**									
Baseline	1146	66.6 (283.6)	7.9 (0.1, 59.9)	571	70.6 (335.5)	8.1 (0.1, 63.0)	571	62.6 (220.4)	7.6 (0.2, 58.0)
12m	987	79.4 (322.8)	15.5 (2.5, 69.0)	498	66.9 (216.7)	15.8 (1.9, 68.5)	489	92.2 (402.9)	15.4 (3.3, 69.0)
24m	488	143.6 (603.6)	19.0 (4.4, 69.2)	244	137.2 (537.0)	18.6 (4.3, 62.3)	244	150.0 (664.5)	20.0 (4.6, 76.0)
36m	931	96.8 (327.7)	17.0 (4.6, 70.0)	463	88.5 (286.3)	17.0 (4.6, 69.8)	468	105.0 (364.1)	17.0 (4.5, 70.5)

Data is presented as mean (SD) or median (IQR Q1, Q3)

SD, standard deviation; IQR, Interquartile range; eGFR, Estimated glomerular filtration rate.

eTable 2: Factors associated with greater decline in eGFR slope (-2.0 ml/min/1.73m2) in the CARRS Trial

				Unadjusted		Adjusted^		Adjusted#	
	**n**	**eGFR slope > -2.0**	**eGFR slope ≤ -2.0**	**Risk ratio (95% CI)**	**p-value**	**Risk ratio (95% CI)**	**p-value**	**Risk ratio (95% CI)**	**p-value**
Overall	1146	313 (27.3)	833 (72.7)						
Age category									
35-44	185	45 (24.3)	140 (75.7)	1.0		1.0		1.0	
45-64	802	226 (28.2)	576 (71.8)	1.16 (0.88, 1.53)	0.298	1.07 (0.77, 1.5)	0.678	1.07 (0.77, 1.49)	0.693
>=65	159	42 (26.4)	117 (73.6)	1.09 (0.76, 1.56)	0.656	1.04 (0.67, 1.62)	0.853	1.03 (0.66, 1.61)	0.881
Sex									
Female	619	176 (28.4)	443 (71.6)	1.0		1.0		1.0	
Male	527	137 (26)	390 (74)	0.91 (0.76, 1.11)	0.357	0.9 (0.7, 1.15)	0.399	0.93 (0.72, 1.2)	0.555
Education									
Up to primary schooling	337	93 (27.6)	244 (72.4)	1.0		1.0		1.0	
Secondary	499	143 (28.7)	356 (71.3)	1.04 (0.83, 1.3)	0.739	1.03 (0.78, 1.36)	0.839	1.03 (0.78, 1.36)	0.829
College graduate & above	303	77 (25.4)	226 (74.6)	0.92 (0.71, 1.19)	0.533	0.9 (0.65, 1.26)	0.543	0.9 (0.65, 1.25)	0.532
Duration of diabetes at baseline									
< 7years	537	131 (24.4)	406 (75.6)	1.0		1.0		1.0	
>=7 years	595	177 (29.7)	418 (70.3)	1.22 (1, 1.48)	0.044	1.08 (0.84, 1.4)	0.543	1.08 (0.84, 1.4)	0.551
CVD at baseline									
No	1068	283 (26.5)	785 (73.5)	1.0		1.0		1.0	
Yes	78	30 (38.5)	48 (61.5)	1.45 (1.08, 1.96)	0.014	1.40 (0.94, 2.07)	0.098	1.38 (0.93, 2.05)	0.111
PVD at baseline									
No	1080	288 (26.7)	792 (73.3)	1.0		1.0		1.0	
Yes	66	25 (37.9)	41 (62.1)	1.42 (1.03, 1.96)	0.034	1.19 (0.77, 1.84)	0.425	1.19 (0.77, 1.83)	0.44
Retinopathy at baseline									
No	1046	275 (26.3)	771 (73.7)	1.0		1.0		1.0	
Yes	100	38 (38)	62 (62)	1.45 (1.1, 1.89)	0.008	1.09 (0.74, 1.6)	0.655	1.10 (0.75, 1.61)	0.63

Neuropathy at baseline									
No	766	203 (26.5)	563 (73.5)	1.0		1.0		1.0	
Yes	380	110 (28.9)	270 (71.1)	1.09 (0.9, 1.33)	0.379	1.01 (0.79, 1.3)	0.922	1.02 (0.79, 1.31)	0.889
Current smoker at baseline									
No	1112	303 (27.2)	809 (72.8)	1.0		1.0		1.0	
Yes	34	10 (29.4)	24 (70.6)	1.08 (0.64, 1.83)	0.777	1.11 (0.58, 2.12)	0.762	1.1 (0.58, 2.11)	0.771
Hba1c % at baseline									
<9	383	101 (26.4)	282 (73.6)	1.0		1.0		1.0	
>=9	763	212 (27.8)	551 (72.2)	1.05 (0.86, 1.29)	0.613	1.00 (0.78, 1.28)	0.975	0.99 (0.78, 1.27)	0.968
Systolic Blood pressure (mmHg)at baseline									
<140	350	98 (28)	252 (72)	1.0		1.0		1.0	
>=140	796	215 (27)	581 (73)	0.96 (0.79, 1.18)	0.728	1.1 (0.79, 1.55)	0.567	1.11 (0.79, 1.56)	0.547
LDL-c (mmHg)at baseline									
<130	604	155 (25.7)	449 (74.3)	1.0		1.0		1.0	
>=130	542	158 (29.2)	384 (70.8)	1.14 (0.94, 1.37)	0.186	1.24 (0.92, 1.68)	0.157	1.25 (0.92, 1.69)	0.15
BMI (Kg/m2) category at baseline									
< 18.5	13	9 (69.2)	4 (30.8)	1.22 (0.52, 2.88)	0.644	1.24 (0.44, 3.48)	0.687	na	
18.5-22.9	171	128 (74.9)	43 (25.1)	1.0		1.0			
23-24.9	183	127 (69.4)	56 (30.6)	1.22 (0.87, 1.71)	0.255	1.17 (0.78, 1.75)	0.451		
>= 25	779	569 (73)	210 (27)	1.07 (0.81, 1.42)	0.63	1.07 (0.76, 1.5)	0.703		
Waist circumference									
(<80 cm (women), < 90 cm (men)	185	140 (75.7)	45 (24.3)	1.07 (0.81, 1.42)	0.63	1			
≥80 cm (women), ≥ 90 cm (men)	961	693 (72.1)	268 (27.9)	1.07 (0.81, 1.42)	1.63	1.15 (0.87, 1.51)	0.328	1.11 (0.79, 1.57)	0.538
Blood pressure medication at baseline									
No	432	121 (28)	311 (72)	1.0		1.0		1.0	
Yes	714	192 (26.9)	522 (73.1)	0.96 (0.79, 1.17)	0.68	0.91 (0.7, 1.19)	0.494	0.9 (0.69, 1.17)	0.432

ACEi/ ARB at baseline									
No	829	233 (28.1)	596 (71.9)	1.0		na		na	
Yes	317	80 (25.2)	237 (74.8)	0.90 (0.72, 1.12)	0.334				
Statins at baseline									
No	467	113 (24.2)	354 (75.8)	1.0		1.0		1.0	
Yes	679	200 (29.5)	479 (70.5)	1.22 (1, 1.48)	0.052	1.15 (0.9, 1.45)	0.264	1.14 (0.9, 1.44)	0.284
Insulin use at baseline									
No	653	160 (24.5)	493 (75.5)	1.0		1.0		1.0	
Yes	493	153 (31)	340 (69)	1.27 (1.05, 1.53)	0.014	1.17 (0.91, 1.49)	0.224	1.18 (0.92, 1.5)	0.199
Urine Albumin to creatinine ratio									
<30	765	572 (74.8)	193 (25.2)	1.0		1.0		1.0	
30-299.99	338	240 (71)	98 (29)	1.15 (0.93, 1.41)	0.187	1.18 (0.92, 1.53)	0.194	1.19 (0.92, 1.54)	0.183
>=300	43	21 (48.8)	22 (51.2)	2.03 (1.48, 2.78)	0	1.73 (1.07, 2.8)	0.026	1.74 (1.07, 2.82)	0.025

adjusted for the treatment group and each of the other covariates listed in the model (excluding waist circumference)
